# Supplementary material for: DAB2 in LGMD R2: a molecular link between disease progression and lipid dysregulation
Source: JCI Insight. 2026 Mar 23;11(6):e200054. doi: 10.1172/jci.insight.200054 (PMC13043104; doi:10.1172/jci.insight.200054)
Supplement: Supplemental data [file jciinsight-11-200054-s089.pdf]

Supplemental

DAB2 in LGMD R2: a molecular link between disease progression and lipid dysregulation

Celine Bruge[1,2], Nathalie Bourg[3], Emilie Pellier[1,2], Quentin Miagoux[1,2], Manon Benabides[1,2], Noella Grossi[1,2], Hassan Hayat[1,2], Margot Jarrige[1,2], Helene Polveche[1,2], Valeria Agostini[3], Anthony Brureau[3], Stéphane Vassilopoulos[4], Teresinha Evangelista[5,6], Gorka Fernández-Eulate[6], Tanya Stojkovic[6], Isabelle Richard[3] and Xavier Nissan[1,2]*

This PDF file includes:

Figure S1. LGMD R2 hiPSC lines maintain pluripotency.
Figure S2. Efficient skeletal myogenic differentiation of LGMD R2 hiPSC lines.
Figure S3. Transcriptomic profiling reveals differential gene expression in LGMD R2 myotubes.
Figure S4. DAB2 transcript is upregulated in LGMD R2 patient muscle biopsies.
Figure S5. DAB2 expression correlates with patient involvement.
Figure S6. Histological characterization of dysferlin-deficient Bla/J mice.
Figure S7. Dysferlin AAV gene therapy restores DAB2 in Bla/J mice.
Figure S8. Lipid accumulation occurs in the most affected muscles of Bla/J mice.
Figure S9. Dysferlin AAV gene therapy normalizes lipid accumulation in Bla/J mice.
Figure S10. siDAB2 treatment normalizes lipid accumulation in LGMD R2 myotubes.
Figure S11. DAB2 is upregulated in dystrophic muscles.

Table S1. Clinical characteristics of patients with dysferlin deficiency.
Table S2. List of antibodies.
Table S3. List of primers.

Supplementary Figure 1

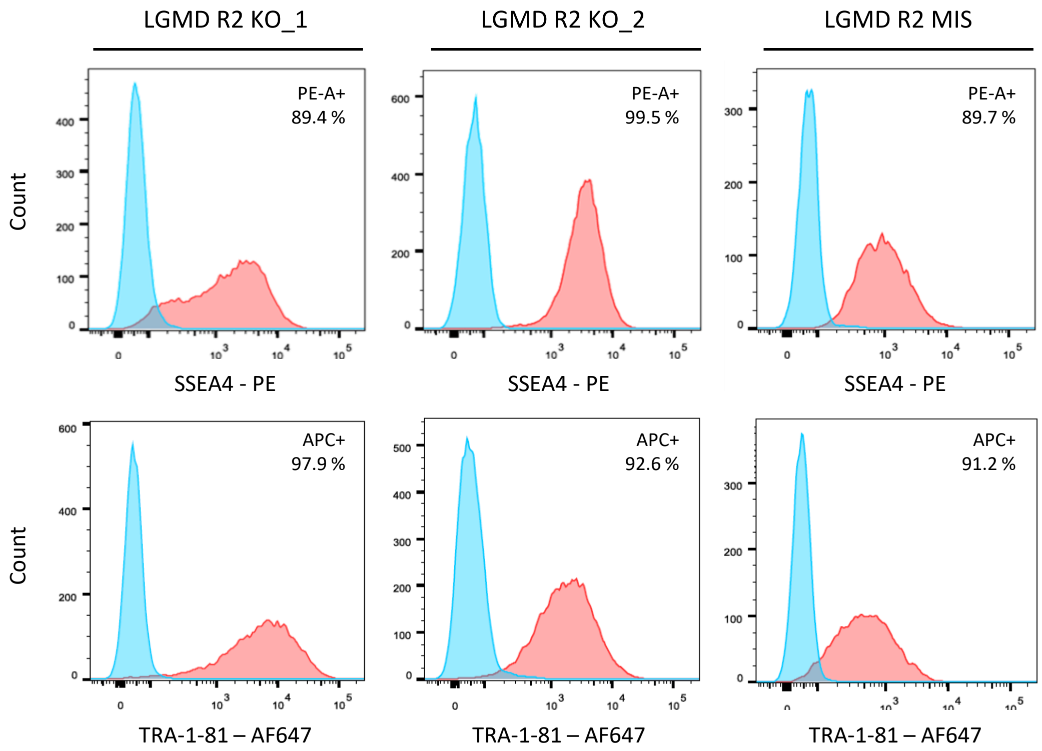

Figure S1. LGMD R2 hiPSC lines maintain pluripotency. Flow cytometry analysis of surface pluripotency markers SSEA4 (PE-A+) and TRA-1-81 (APC+) in three LGMD R2 hiPSC lines. Blue histograms represent isotype controls, and red histograms represent experimental samples.

Supplementary Figure 2

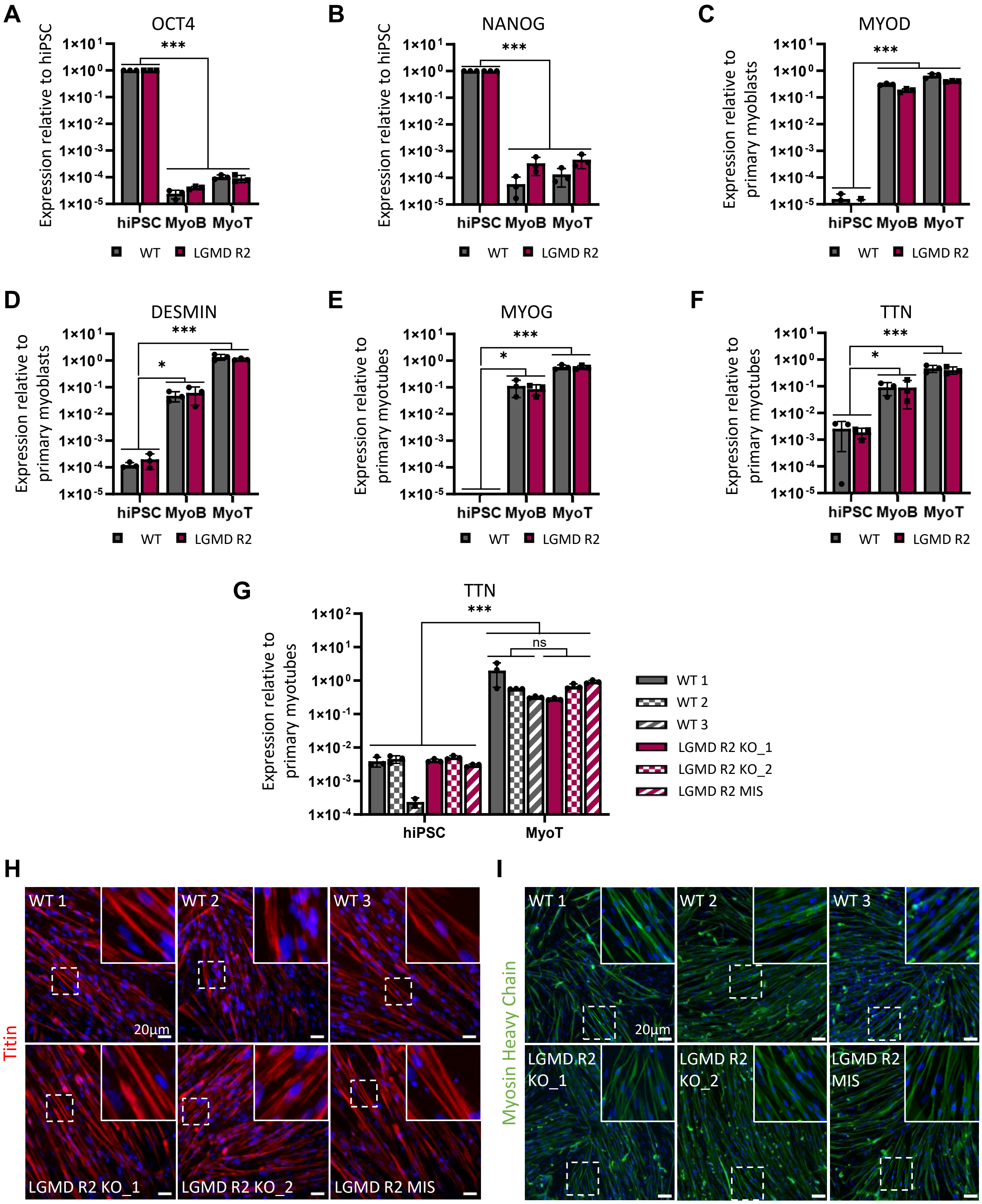

Figure S2. Efficient skeletal myogenic differentiation of LGMD R2 hiPSC lines. (**A-F**) mRNA expression levels of pluripotency marker OCT4 (**A**) and NANOG (**B**), and myogenic markers MYOD (**C**), DESMIN (**D**), MYOG (**E**), and TTN (**F**) at day 0 (hiPSC), day 17 (myoblasts), and day 24 (myotubes). Gene expression was normalized to hiPSC, primary myoblasts, or primary myotubes, respectively. Data are shown as mean ± SD of three control (grey) or three LGMD R2 (pink) lines (n=3). *p ≤ 0.05, ***p ≤ 0.001 (Nonparametric Mann-Whitney test). **(G**) TTN expression measured by qPCR at day 0 (hiPSC) and day 24 (myotubes), normalized to primary myotubes. Data represent mean ± SD of three skMC differentiations per cell line (n=3). *p ≤ 0.05 hiPSC vs. MyoT group (Nonparametric Mann-Whitney test); ns p > 0.05 WT vs. LGMD R2 group at myotube stage (Nonparametric Mann-Whitney test). Characterization of titin (red) (**H**) and myosin heavy chain (MHC) (**I**) expression by immunostaining in control and LGMD R2 hiPSC-derived myotubes (day 24). Nuclei were stained with Hoechst (blue). White boxes indicate magnified areas. Scale bar: 20 µm.

Abbreviations: hiPSC, human induced pluripotent stem cells; MyoB, myoblasts; MyoT, myotubes.

Supplementary Figure 3

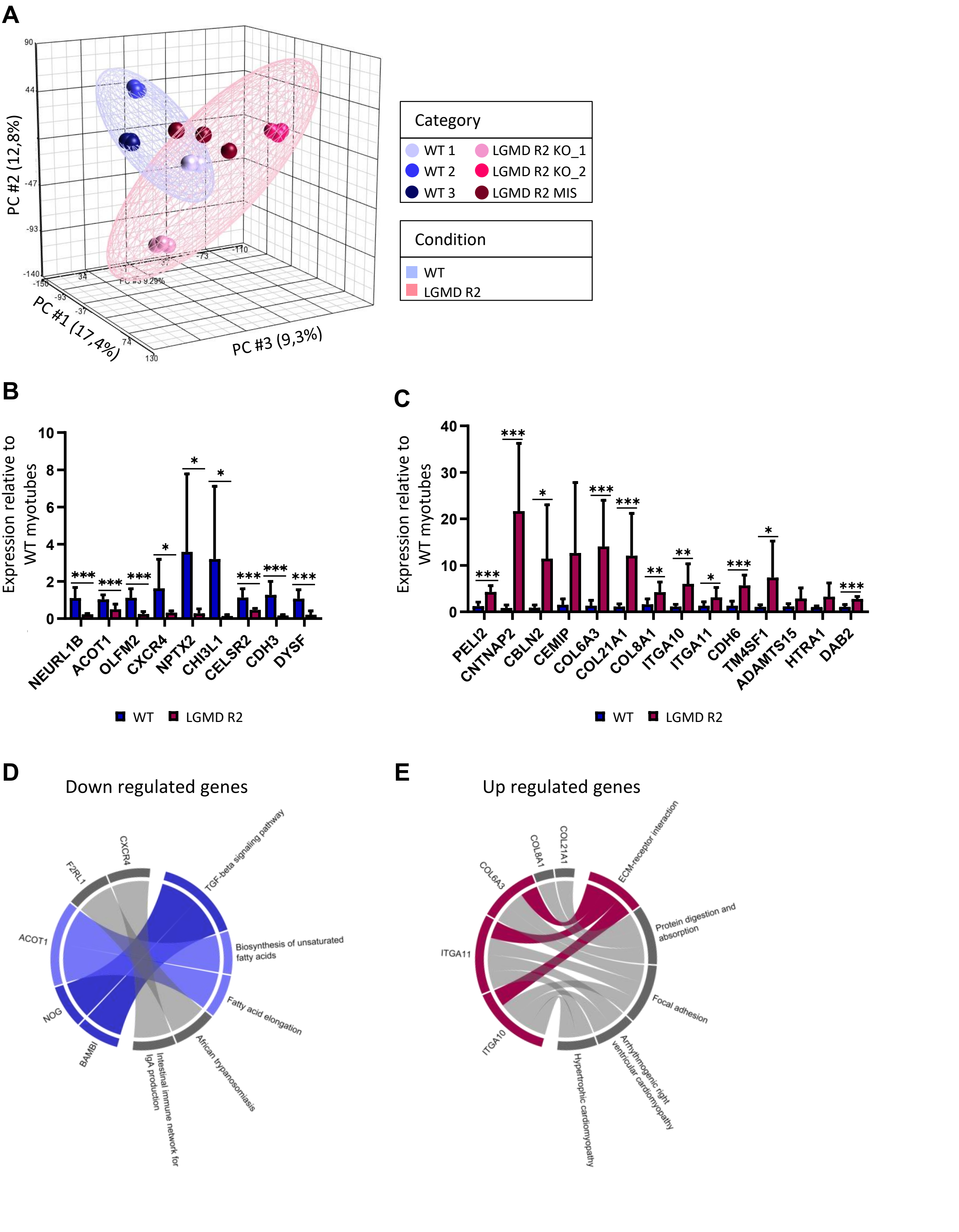

Figure S3. Transcriptomic profiling reveals differential gene expression in LGMD R2 myotubes. (**A**) Principal component analysis (PCA) of transcriptome profiles obtained from myotubes derived from three control (blue) and three LGMD R2 (pink) lines. Each point represents an independent skMC differentiation (n=3 per cell line). Samples with similar gene expression profiles cluster together. (**B-C**) Validation of 23 out of 52 differentially expressed genes by quantitative PCR. Genes down-regulated (**B**) or up-regulated (**C**) in LGMD R2 myotubes are shown. Gene expression was normalized to the mean of myotubes derived from three control lines. Data are represented as mean ± SD of three myogenic differentiations from three control lines (blue) or three LGMD R2 lines (pink) (n=9). $^{*}p \leq 0.05$, $^{**}p \leq 0.01$, $^{***}p \leq 0.001$ (Nonparametric Mann-Whitney test). (**D-E**) KEGG 2021 Human enrichment analysis of 13 significantly down-regulated genes (**D**) and 39 significantly up-regulated genes (**E**) in LGMD R2 myotubes. The top five enriched pathways ($p < 0.05$) are shown. Pathways relevant to pathology are highlighted in blue (down-regulated) or pink (up-regulated). Lines connect genes to the pathways in which they are involved.

Supplementary Figure 4

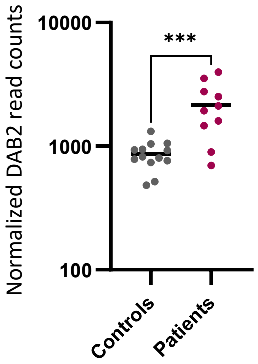

Figure S4. DAB2 transcript is upregulated in LGMD R2 patient muscle biopsies. Analysis of DAB2 expression (read counts) from a previously published Affymetrix transcriptomic dataset comparing 10 muscle biopsies from LGMD R2 patients (pink) and 13 unaffected individuals (grey). Each dot represents an individual biopsy. ***p ≤ 0.001 (Unpaired t-test with Welch's correction).

Supplementary Figure 5

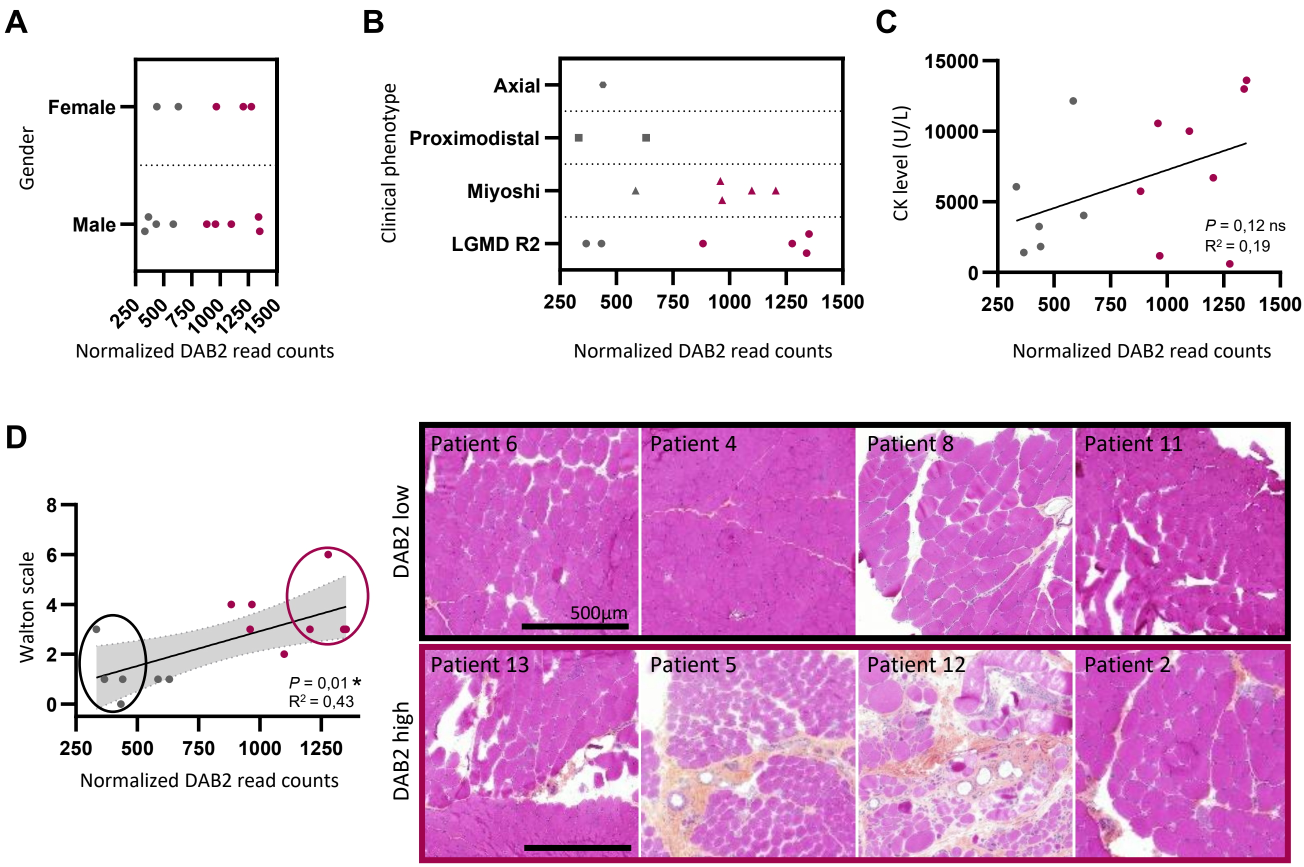

Figure S5. DAB2 expression correlates with patient involvement. Analysis of DAB2 expression in dysferlin-deficient muscle biopsies according to gender (**A**), clinical phenotype (**B**), or creatine phosphokinase levels (**C**). Each dot represents an individual biopsy. DAB2 expression is color-coded from low (grey) to high (pink). No significant correlations were observed (ns, $p > 0.05$, Pearson correlation analysis). (**D**) Visualization of DAB2 expression relative to patient involvement based on the Walton scale (poorly involved 0 ≤ Walton scale ≤ 10 severely involved). Representative HPS-stained muscle sections are shown for patients with the lowest (grey circle) and highest (pink circle) DAB2 expression. Scale bar = 500 µm. *$p \leq 0.05$ (Pearson correlation analysis). Abbreviation: CK, creatine kinase.

Supplementary Figure 6

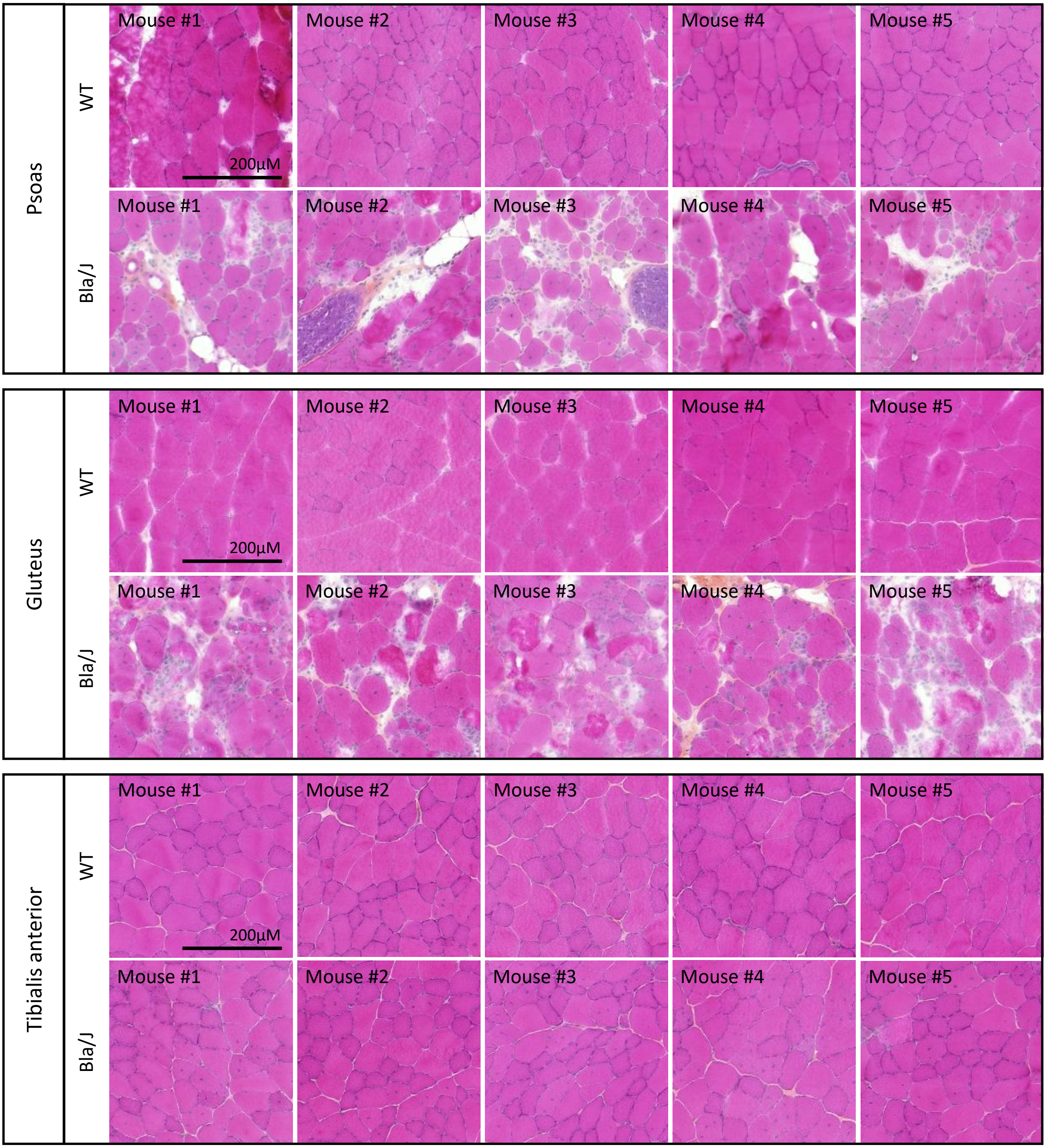

Figure S6. Histological characterization of dysferlin-deficient Bla/J mice. Comparative analysis of psoas, gluteus and tibialis anterior muscles from six-month-old control (WT) and Bla/J mice using HPS staining. Scale bar = 200 µm.

Supplementary Figure 7

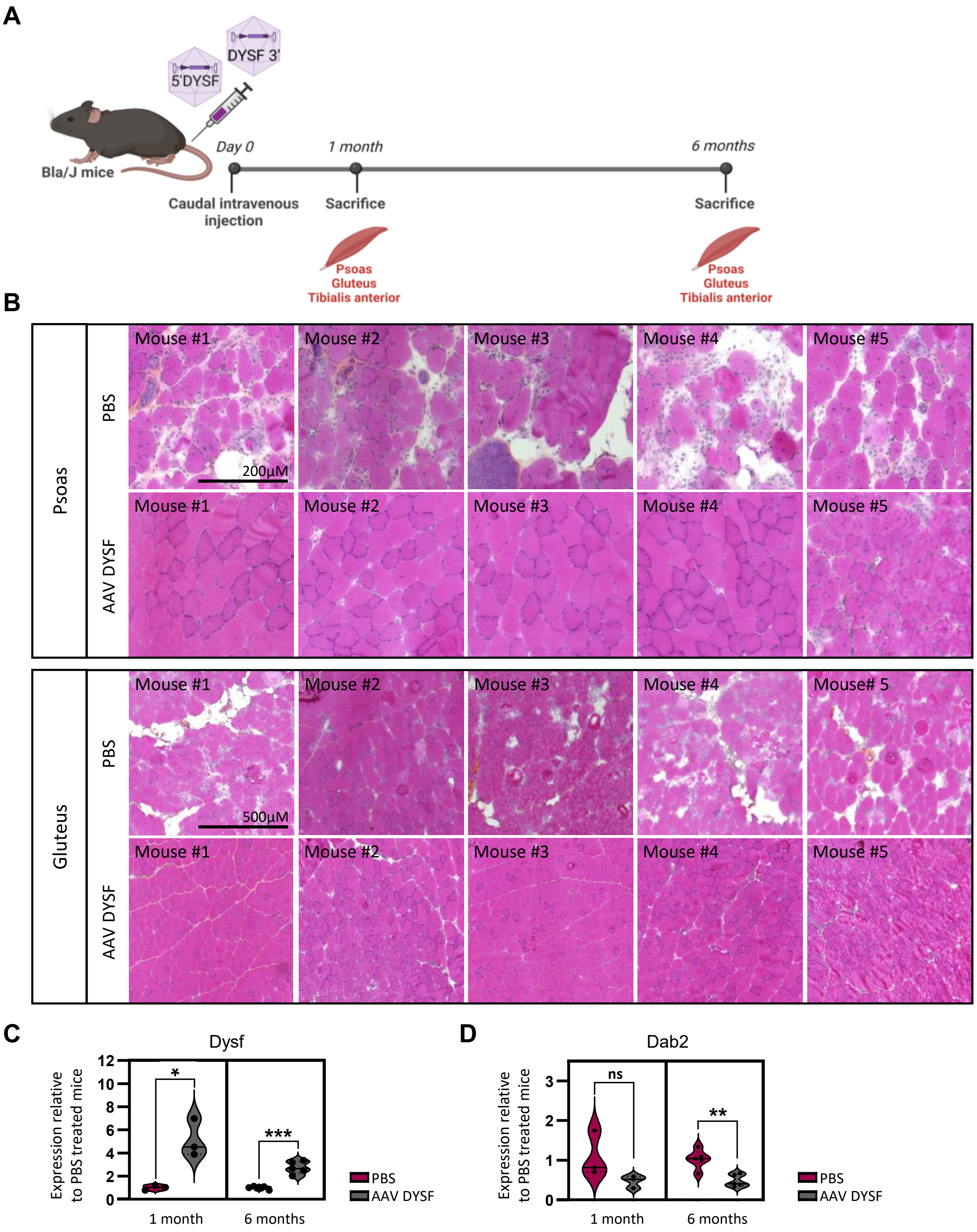

Figure S7. Dysferlin AAV gene therapy restores DAB2 in Bla/J mice. (**A**) Schematic representation of the gene therapy protocol for one-month-old Bla/J mice using a dual AAV vector to restore full-length dysferlin. Mice received a single treatment; protocol duration is indicated. Created with BioRender.com. (**B**) Representative HPS-stained sections of psoas and gluteus muscles from Bla/J mice six months after treatment with PBS or dual AAV dysferlin rescue (AAV DYSF). Scale bar = 200 or 500 µm. (**C–D**) qPCR analysis of Dysf (**C**) and Dab2 (**D**) expression in gluteus muscle after one or six months of treatment. Expression was normalized to PBS-treated mice. Data represent mean ± SD of mice per condition (n = 3–5). *p ≤ 0.05 or **p ≤ 0.01, ***p ≤ 0.001 (Unpaired t-test with Welch's correction).

Supplementary Figure 8

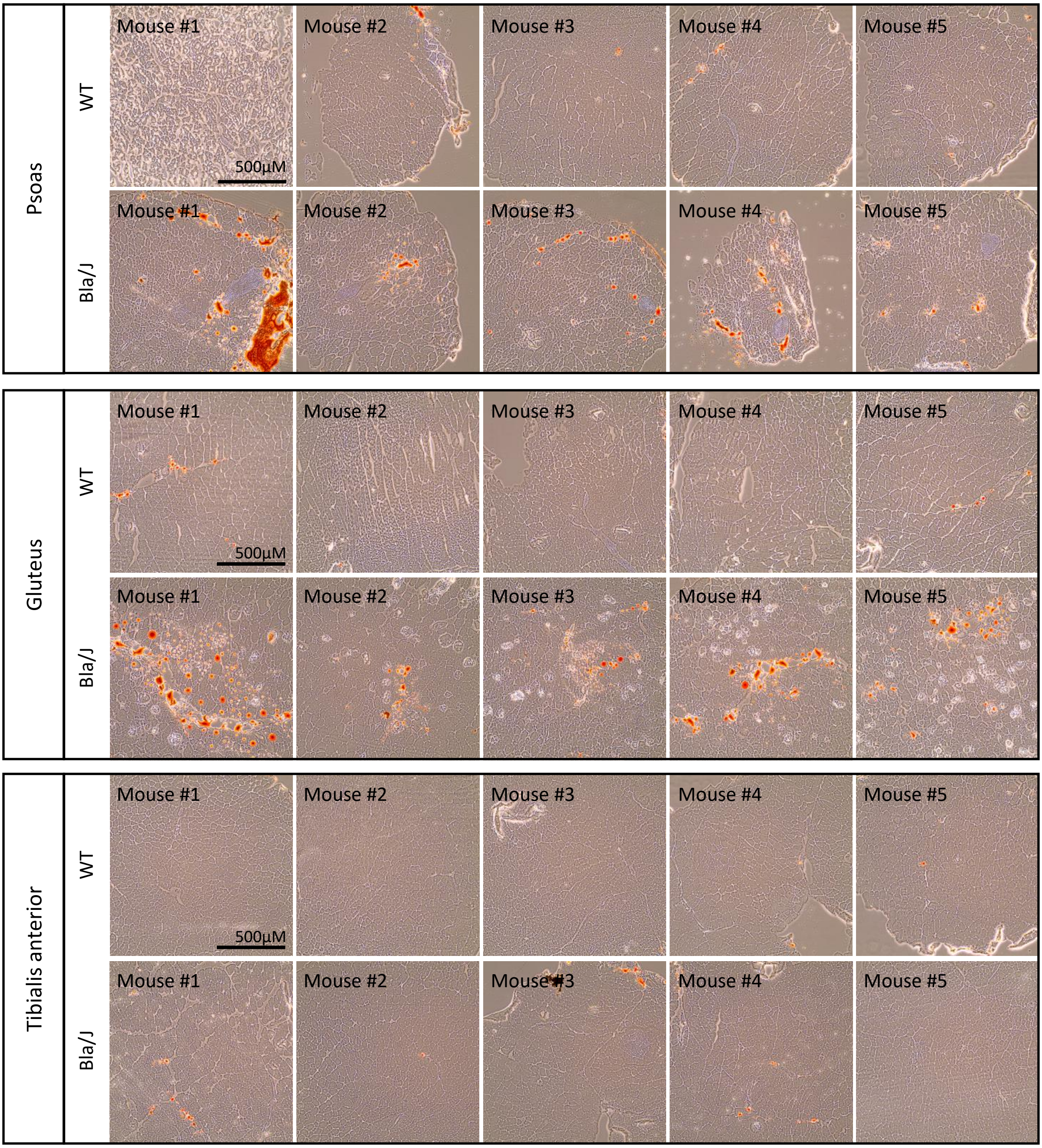

Figure S8. Lipid accumulation occurs in the most affected muscles of Bla/J mice. Representative Oil Red O-stained sections of psoas, gluteus, and tibialis anterior muscles from six-month-old WT and Bla/J mice. Scale bar = 500 µm.

Supplementary Figure 9

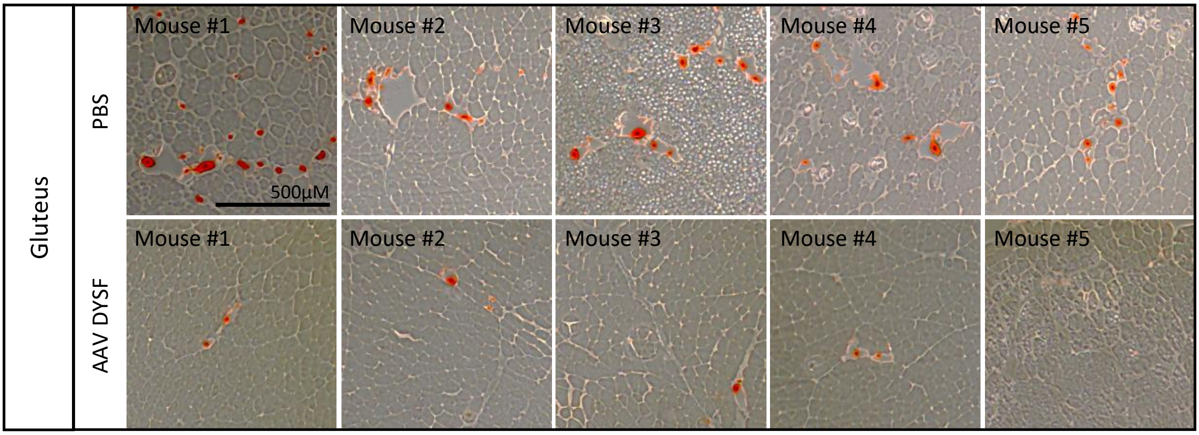

Figure S9. Dysferlin AAV gene therapy normalizes lipid accumulation in Bla/J mice. Representative Oil Red O-stained sections of gluteus muscles from Bla/J mice six months after treatment with PBS or dual AAV dysferlin rescue (AAV DYSF). Scale bar = 500 µm.

Supplementary Figure 10

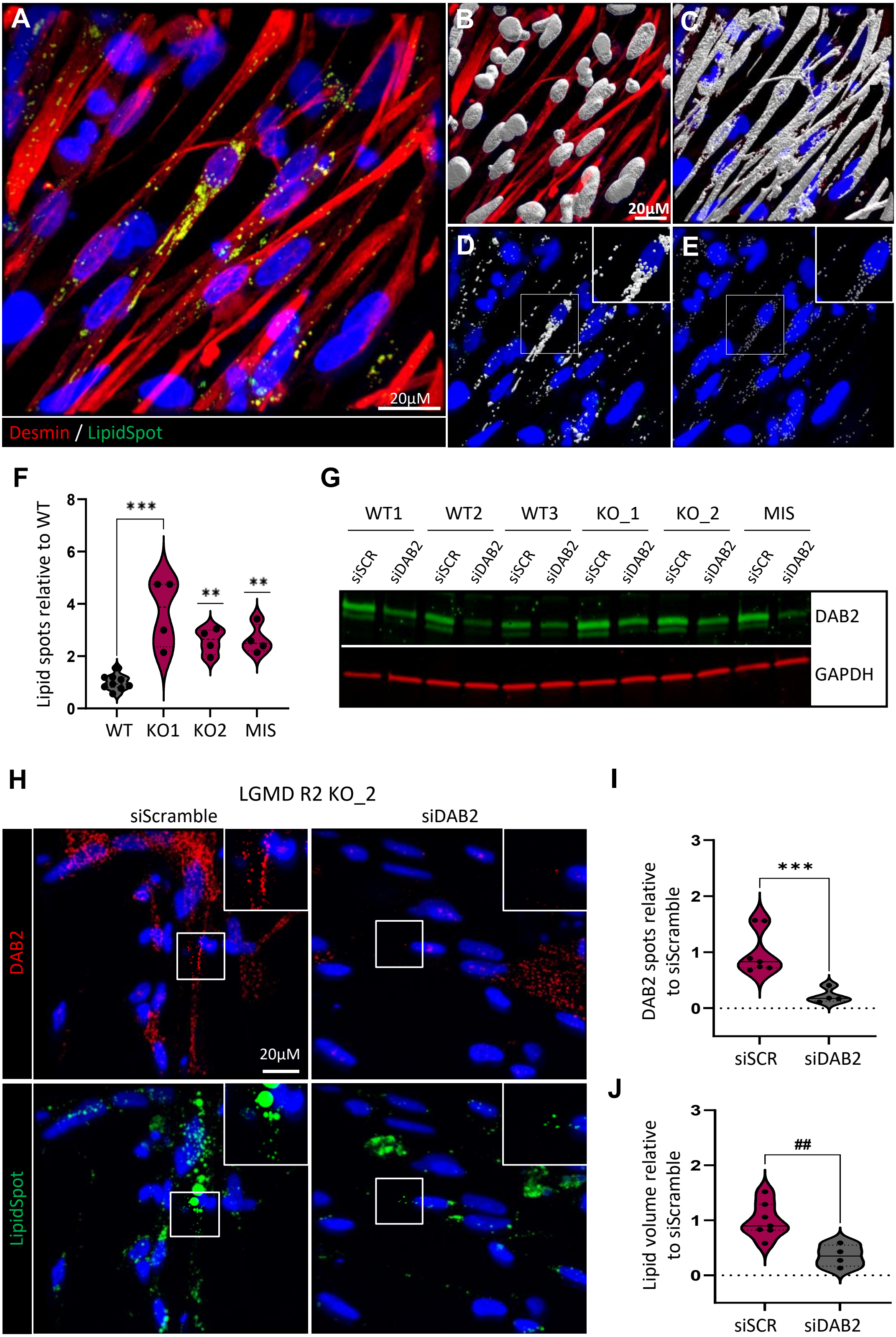

Figure S10. siDAB2 treatment normalizes lipid accumulation in LGMD R2 myotubes.
(**A-E**) Immunostaining of lipid droplets (LipidSpot, green) in hiPS-derived myotubes (desmin, red) supplemented with fatty acids for 48 hours (**A**). Nuclei were counterstained with Hoechst (blue). Imaris detection masks were used to identify nuclei (**B**), myotubes (**C**), and lipid droplets for quantification of lipid volume (**D**) and lipid droplet number (**E**). White boxes indicate magnified regions. Scale bar = 20 µm. (**F**) Quantification of lipid droplet number in WT and LGMD R2 hiPSC-derived myotubes. Data represent mean ± SD of a representative experiment from *n*=3 experiments. **p ≤ 0.01, ***p ≤ 0.001 (One-way ANOVA, Dunnett's multiple comparisons test). (**G-J**) Analysis of siDAB2 treatment on lipid accumulation in fatty acid-supplemented myotubes. (**G**) Representative immunoblot of human DAB2 P96 isoform expression (green) in healthy and LGMD R2 myotubes treated for 48 hours with siRNA targeting DAB2. GAPDH is a loading control. (**H**) Immunostaining of DAB2 (red) and lipid droplets (green) in LGMD R2 KO_2 hiPSC-derived myotubes after 48 hours of treatment with siScramble (left) or siDAB2 (right). White boxes indicate magnified regions. Scale bar = 20 µm. Associated quantification of DAB2 droplet number (**I**) and lipid volume (**J**) in LGMD R2 KO_2 hiPSC-derived myotubes after treatment with siScramble (pink) or siDAB2 (grey). Data represent mean ± SD of a representative experiment from *n*=3 experiments. ##p ≤ 0.01, ***p ≤ 0.001 (Unpaired t test; transformed data for lipid volume). Abbreviation: siSCR, siScramble.

Supplementary Figure 11

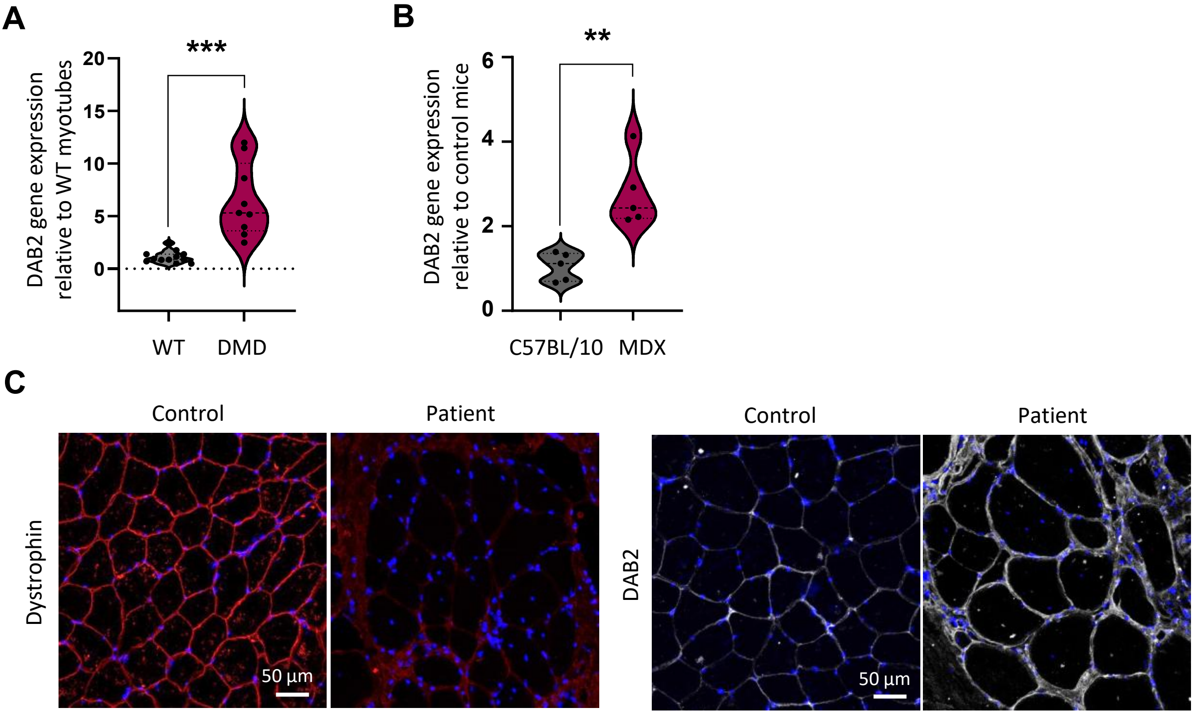

Figure S11. DAB2 is upregulated in dystrophic muscles. (**A**) DAB2 gene expression in DMD hiPSC-derived myotubes relative to WT controls. Each dot represents an independent differentiation replicate (n = 3 per cell line). ***p ≤ 0.001 (unpaired t-test on log-transformed values). (**B**) DAB2 gene expression in DMD mouse model (MDX) compared with C57BL/10 controls. Each dot represents one mouse (n = 5). **p ≤ 0.01 (unpaired t-test). (**C**) Immunostaining of dystrophin (red) and DAB2 (white) in muscle fibers from control and DMD patient biopsies. Nuclei are counterstained with Hoechst (blue). Scale bar = 50 µm. Abbreviations: DMD, Duchenne muscular dystrophy.

Supplementary Table 1

Table S1. Clinical characteristics of patients with dysferlin deficiency.

Patient	Gender	Muscle biopsy	Clinical phenotype	Genetic mutation	Dysferlin expression (wb)	Age at disease onset (years)	Biopsy age (years)	Diagnostic at biopsy	CK level (IU/L)	Walton score
1	Male	Deltoide	LGMD R2	HTZ: c.2998T>C / c.4795-1G>C	absent	16	31	Dystrophic	5753	IIII
2	Male	Deltoide	LGMD	HMZ: c.5341-2A>C	absent	22	23	Dystrophic	13618	III
3	Male	Deltoide	Miyoshi	HTZ: c.2643+1G>A / c.4577A>C	absent	18	35	Dystrophic	10561	III
4	Male	Deltoide	LGMD R2	HTZ: c.6017G>A / Ø	absent	3	15	Aspecific myopathic	1404	I
5	Female	Finger expander	LGMD R2	HMZ: c.6044T>C	absent	17	36	Dystrophic	605	VI
6	Male	Deltoide	Proximodistal	HMZ: del exon 55	absent	30	35	Dystrophic	6072	III
7	Male	Anterior hamstring	Miyoshi myopathy	HTZ: c.3517dupT / c.5871_5872delGT	absent	19	20	Dystrophic	12147	I
8	Male	Deltoide	LGMD R2	HTZ: c.757C>T / c.5813-5821del	trace	35	33	Aspecific myopathic	3247	0
9	Female	Deltoide	Miyoshi	HMZ: c.5314-5318del	absent	37	56	Aspecific myopathic	1167	IIII
10	Male	Deltoide	Miyoshi	HTZ: c.1911C>A / c.2641A>C	trace	17	20	Dystrophic	10000	II
11	Female	Deltoide	Axial	HTZ: c.906+4A>G / c.1813C>T	absent	52	55	Aspecific myopathic	1828	I
12	Male	Deltoide	LGMD R2	HTZ: del exon 5 / del exon 5	absent	23	30	Dystrophic	13000	III
13	Female	Deltoide	Miyoshi	HTZ: c.20T>C / c.2810C>T	trace	18	24	Dystrophic	6710	III
14	Female	Deltoide	Proximodistal	HTZ: del exon 5 / c.906+4A>G	absent	37	41	Dystrophic	4043	I

Patient	Gender	Muscle biopsy	Clinical phenotype	Genetic mutation	Dysferlin expression (wb)	Age at disease onset (years)	Biopsy age (years)	Diagnostic at biopsy	CK level (IU/L)	Walton score
1	Female	Deltoide			present	CONTROL	25			
2	Male	Deltoide			present	CONTROL	32			

Supplementary Table 2

Table S2. List of antibodies.

Application	Antibodies	Supplier	Reference	Dilution
Flow cytometry	TRA-1-81 AF647	Biolegend	330706	1:25
	AF647 Isotype control	Biolegend	401618	1:250
	SSEA4 PE	Miltenyi	130-122-914	1:50
	PE Isotype control	Miltenyi	130-113-462	1:50
Immunostaining	α-actinin	Sigma	A7811	1 :1 000
	Dab2	Abcam	ab33441	1 :200
	Desmin	R&D systems	AF3844	1 :200
	Dysferlin	Abcam	ab124684	1 :200
	Dysferlin	Novocastra	HAMLET-CE	1 :50
	LipidSpot 488	Biotium	70065	1 :1000
	Myosin heavy chain	DSHB	3ea	1 :50
	Titin	US Biological	T5650	1 :100
	Donkey anti-mouse AF488	Invitrogen	A-21202	1 :1 000
	Donkey anti-rabbit AF488	Invitrogen	A-21206	1 :1 000
	Donkey anti-mouse AF555	Invitrogen	A-31570	1 :1 000
	Donkey anti-rabbit AF555	Invitrogen	A-31572	1 :1 000
	Donkey anti-goat AF555	Invitrogen	A-21432	1 :1 000
Western Blot	Actin	Sigma	A2066	1 :300
	Dysferlin	Novocastra	HAMLET-CE	1 :500
	Dab2	Abcam	ab33441	1 :500
	Dab2	BD Biosciences	610465	1 :100
	GAPDH	Thermofisher	39-8600	1 :500
	IRDye® 680RD Donkey anti-Rabbit	Li-Cor	926-68073	1 :10 000
	IRDye® 800CW Donkey anti-Rabbit	Li-Cor	926-32213	1 :5 000
	IRDye® 680RD Donkey anti-Mouse	Li-Cor	926-68072	1 :10 000
	IRDye® 800CW Donkey anti-Mouse	Li-Cor	926-32212	1 :5 000

Supplementary Table 3

Table S3. List of primers.

List of human primers.

Gene	Forward	Reverse
ACOT1	ATCTGGAGTACTTTGAAGAAGCTGT	CCAACTCCTGGACCTTTTACCTCA
ADAMTS15	AACAAGCACAGGGTGGATGG	TGCAGGATCGGTATTTCACCC
CBLN2	ACCATCCAGGTCAGTTTAATGC	AACACCAAGAAGCCCGAGAA
CDH3	CAGCTACCGCATCCTGAGAG	GTGGGAGGGCTTCCATTGTC
CDH6	CCCGATTCCCCCAGAGTACAT	CGTCGCTGGCTTTGATTCTG
CELSR2	ATGTGTGTGACCTGAACCCG	GGCTGGTCAATCCTGGTCTC
CEMIP	ACTCAGGGCTGTTGTTCCTG	TCGGTGAACTTGGGGTAAGC
CHI3L1	AAGAACAGGAACCCCAACCTG	TGCTGTTTGTCTCTCCGTCC
CNTNAP2	ACCCCAGATGGAAGCCCTTA	GATCTGTGCAGTTGCGTTCG
COL21A1	CCTGGAACACCGGGATCTAA	TGCTCCTGGTTCTCCCTTGA
COL6A3	TGCGGGAAGCAGGATAACAG	CATCATCCCCAGACCTGTCG
COL8A1	CCTGGGTCAGCAAGTACCTC	TGGTATTTCTTTGCCTTTCTTGGG
CXCR4	TGGTCTATGTTGGCGTCTGG	GTCATTGGGGTAGAAGCGGT
DAB2	GACCAGATGGATTTGTTTGGGG	GAACAGGAGGTGATGCCGTT
DESMIN	ATTGGAGGACCGATTTGCC	TCACCGTCTTCTTGGTATGGA
HTRA1	TGTCACTCACGTCCAGCAAA	GACATCATTGGCGGAGACCA
ITGA10	GGGGGAGCAGATTGGTTCAT	GTTCCTTGGAGGGTCAGCAA
ITGA11	AATAAGTGGCTGGTCGTGGG	GTGACCCTTCCCAGGTTGAG
MYOD	GGGGCTAGGTTCAGCTTTCT	CTACATTTGGGACCGGAGTG
MYOG	TAAGGTGTGTAAGAGGAAGTCG	CCACAGACACATCTTCCACTGT
NANOG	CAAAGGCAAACAACCCACTT	TCTGCTGGAGGCTGAGGTAT
NEURL1B	GTGAGTCCGTCCAGTTGAGG	GGGACTCGGATAAGACAGCG
NPTX2	TTGGACAAGAGCAGGACACC	GAGCAGTTGGCGATGTTGAC
OCT4	CCTCACTTCACTGCACTGTA	CAGGTTTTCTTTCCCTAGCT
OLFM2	TGGAGCAGTACAAGGCAGAC	ATACCCGTAGGCACCCATCT
PELI2	CGGATATGTTTCAGGTGGGC	CTGTGTGATCTGGGCTTCGT
TM4SF1	GCAAACGATGTGCGATGCT	GGCCGAGGGAATCAAGACAT
18S	TCTTCAGTCCGCTCCAGGTCT	GAGGATGAGGTGGAACGTGT

List of mouse primers.

Gene	Forward	Reverse
DYSF	GGTGTTCGAGAACCAGACCC	GCTGTACTCCCAGCCTTGTT
DAB2	CTCAGGGACAACACAAGCAA	AAATTGATGCTGGCCTTCAC
PO	CTCCAAGCAGATGCAGCAGA	ATAGCCTTGCGCATCATGGT
