# Supplementary material for: DAB2 in LGMD R2: a molecular link between disease progression and lipid dysregulation
Source: JCI Insight. 2026 Mar 23;11(6):e200054. doi: 10.1172/jci.insight.200054 (PMC13043104; doi:10.1172/jci.insight.200054)

Full unedited gel for Figure 1B

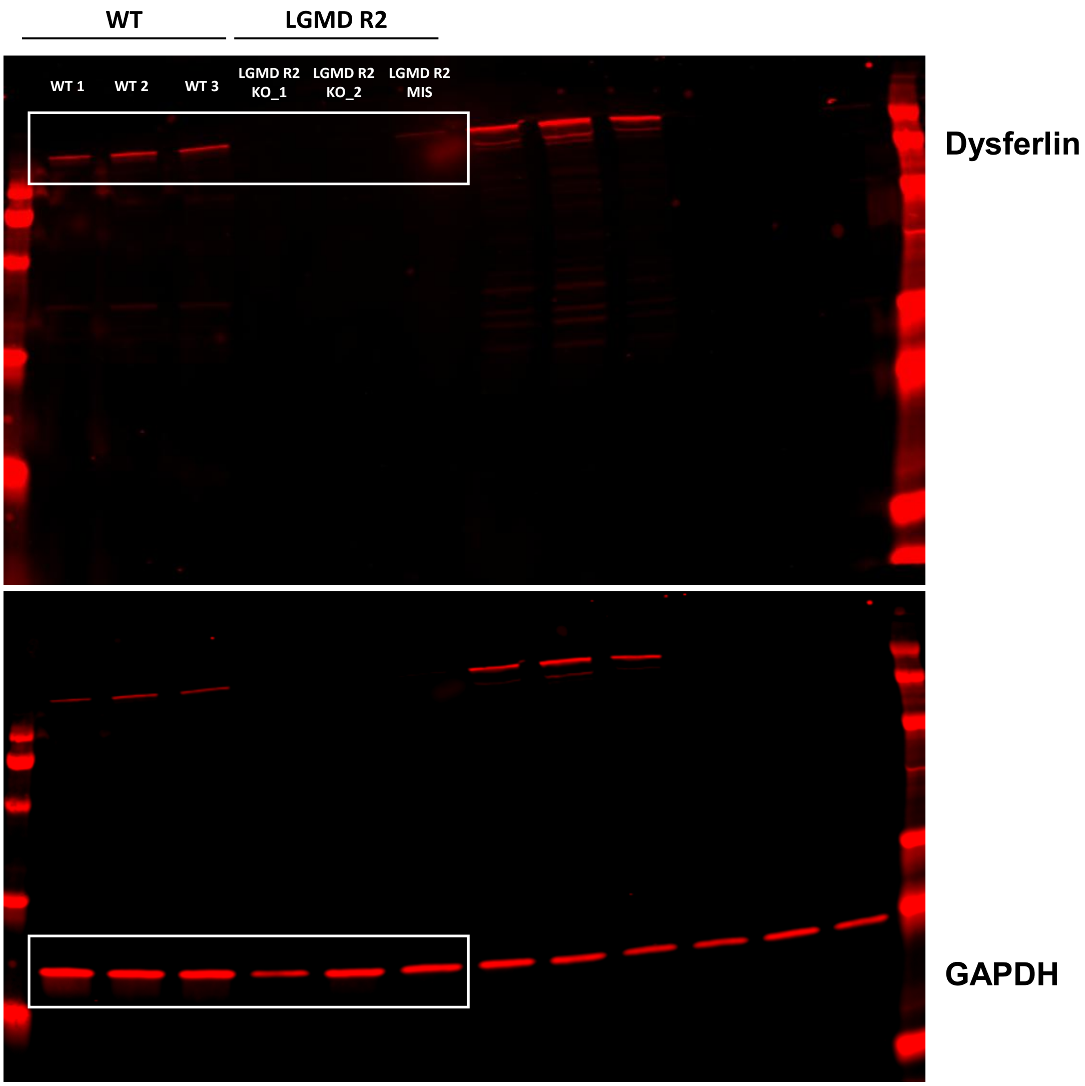

Full unedited gel for Figure 1C

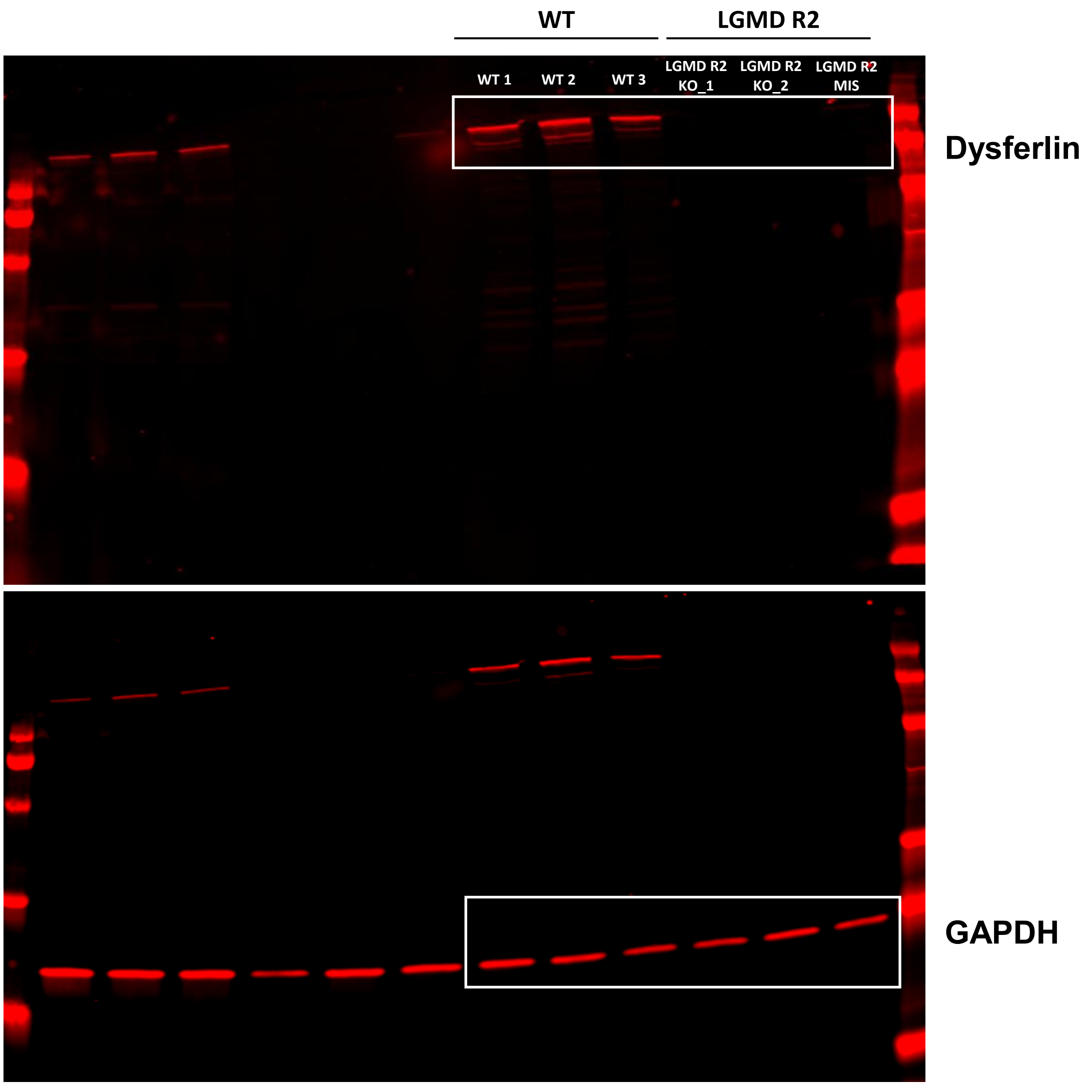

Full unedited gel for Figure 3B

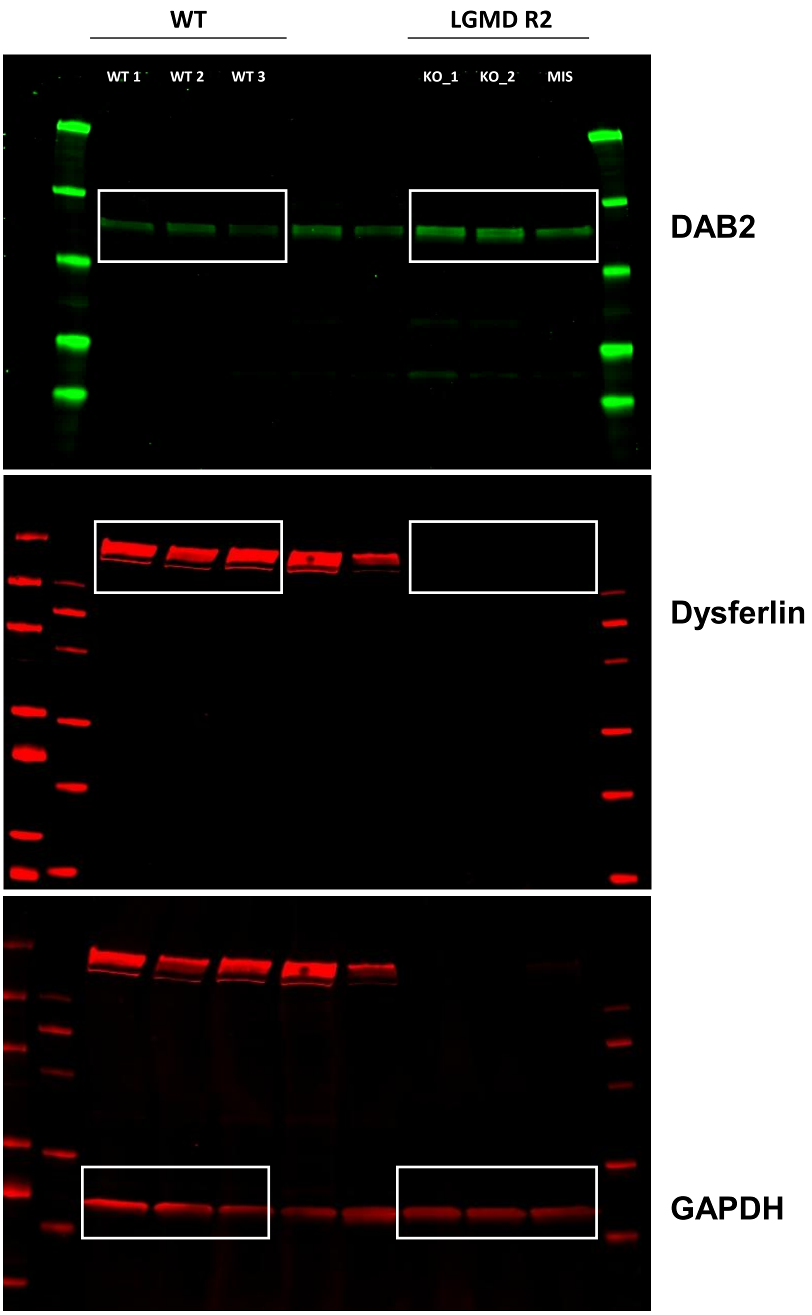

Full unedited gel for Figure 4D

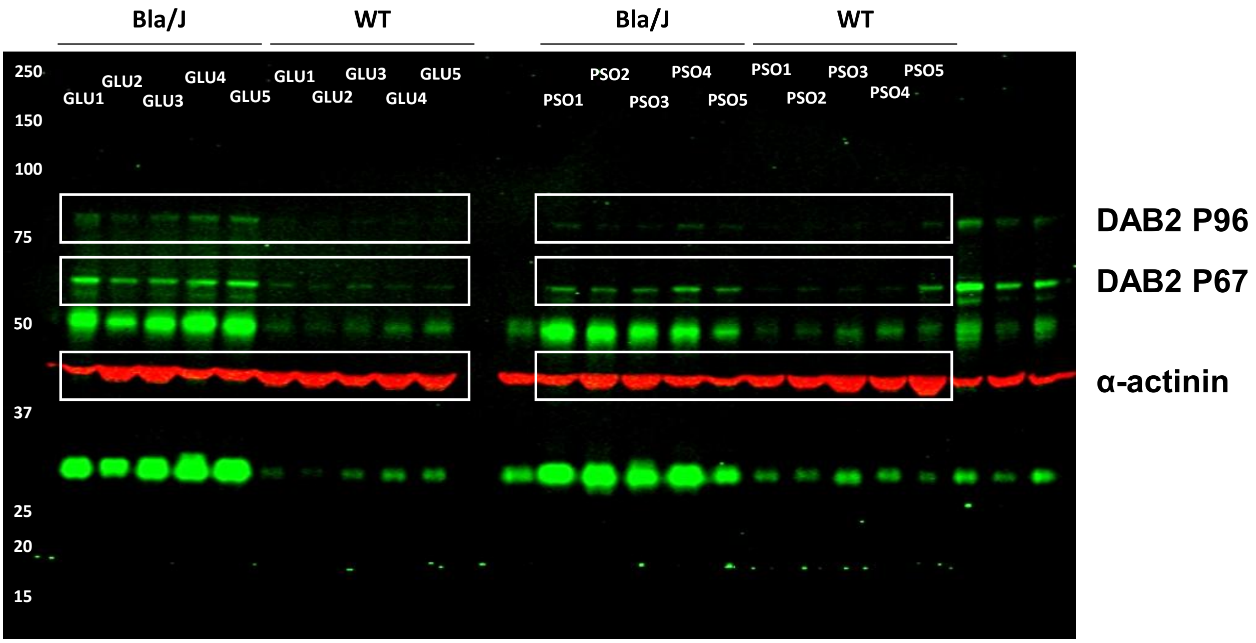

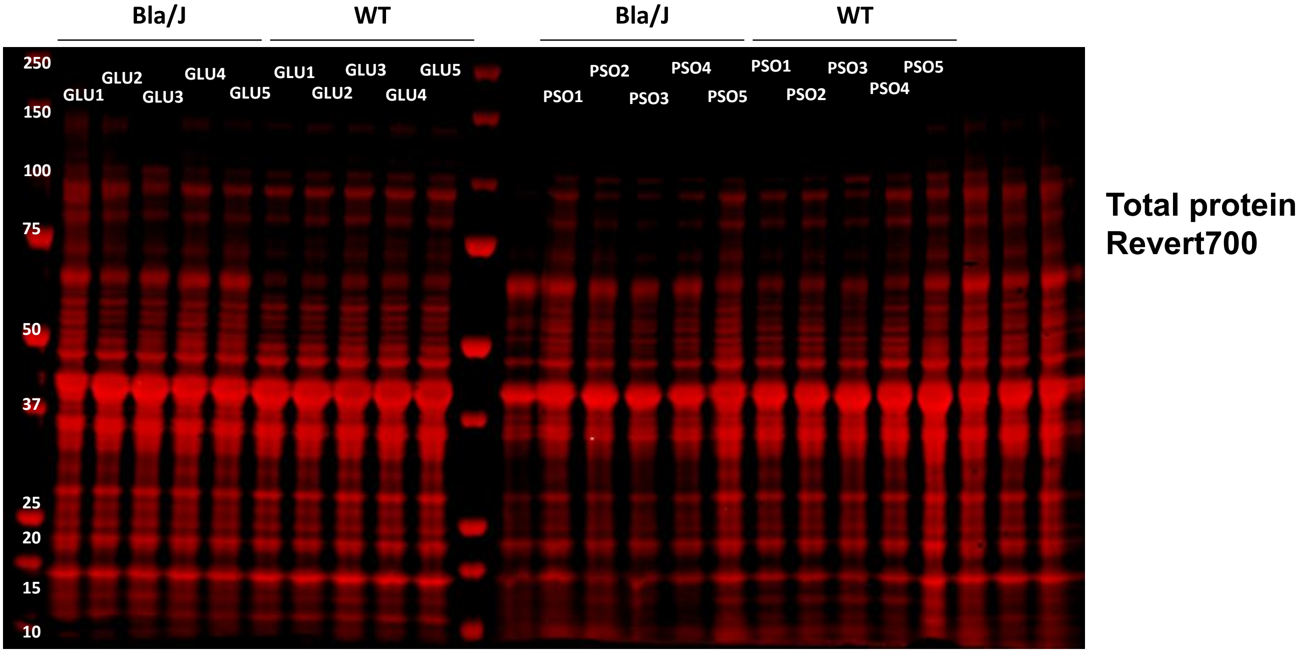

Full unedited gel for Supplementary Figure 10G

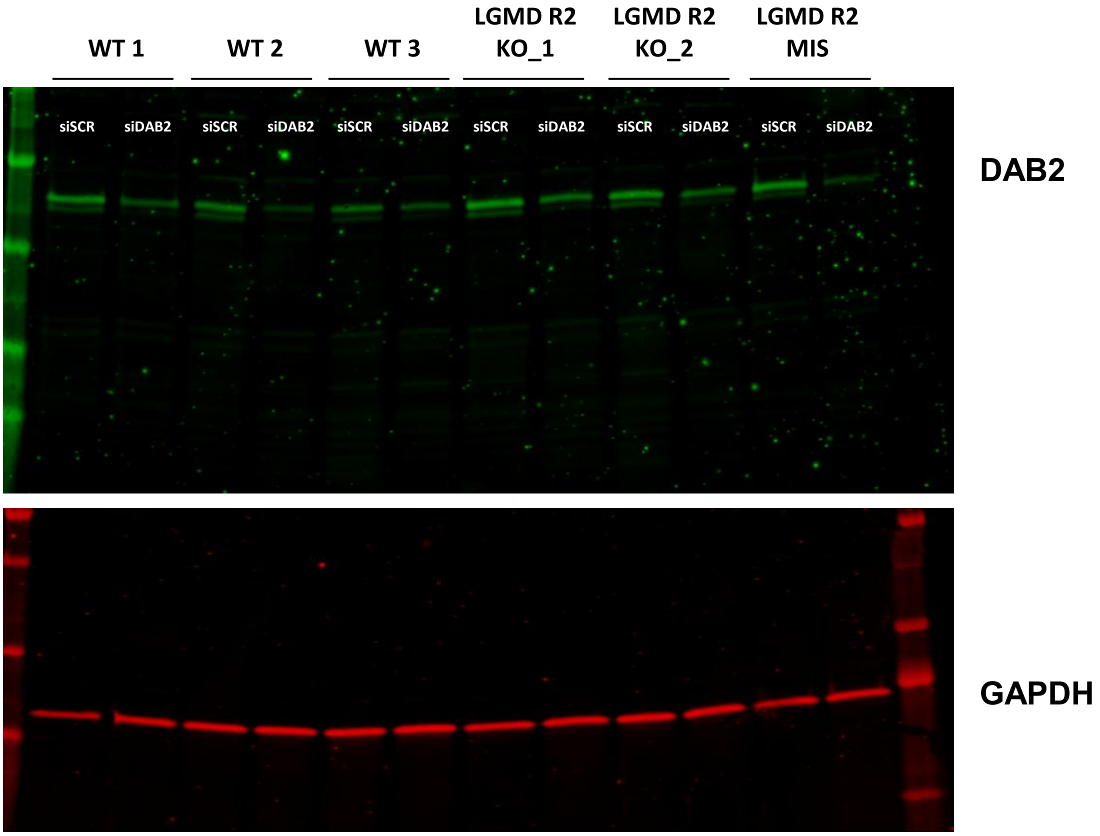

Supplement: Unedited blot and gel images [file jciinsight-11-200054-s090.pdf]
